# Retrospective comparison of axial pattern and subdermal plexus flaps in canine reconstructive surgery: Surgical outcomes, complications, and factors associated with wound dehiscence

**DOI:** 10.14202/vetworld.2026.2393-2405

**Published:** 2026-06-10

**Authors:** Nithida Boonwittaya, Pissamai Noonoi, Suwadee Prawadwin, Naris Thengchaisri

**Affiliations:** 1 Surgery Unit, Kasetsart University Veterinary Teaching Hospital, Faculty of Veterinary Medicine, Kasetsart University, Bangkok 10900, Thailand; 2 Department of Companion Animal Clinical Sciences, Faculty of Veterinary Medicine, Kasetsart University, Bangkok 10900, Thailand

**Keywords:** axial pattern flap, canine reconstructive surgery, complications, dogs, postoperative outcomes, subdermal plexus flap, wound dehiscence, wound reconstruction

## Abstract

**Background and Aim:**

Axial pattern flaps (APFs) and subdermal plexus flaps (SPFs) are widely used reconstructive techniques for managing extensive skin defects in dogs. Although APFs are traditionally considered more reliable because of their direct vascular supply, comparative clinical data between APFs and SPFs remain limited. This study retrospectively compared surgical outcomes, postoperative complications, and factors associated with wound dehiscence in dogs undergoing reconstructive surgery using APFs or SPFs.

**Materials and Methods:**

Medical records of dogs that underwent reconstructive skin flap surgery at the Kasetsart University Veterinary Teaching Hospital, Thailand, between July 2013 and July 2025 were retrospectively reviewed. Data collected included flap type, patient demographics, hematologic and biochemical parameters, surgical indications, wound locations, drainage methods, postoperative complications, and healing outcomes. A total of 85 skin flaps from 78 dogs were analyzed, including 32 APFs and 53 SPFs. Wound dehiscence and postoperative complications were compared between groups using appropriate statistical analyses.

**Results:**

Wound dehiscence occurred in 31/85 flaps (36.5%), with no significant difference between APFs (37.5%) and SPFs (35.8%) (p = 1.000). Overall postoperative complications were observed in 61% of flaps and were comparable between APFs and SPFs (65.6% vs. 58.5%, p = 0.513). Mass removal was the primary indication for reconstruction (81.2%). Distal limb reconstructions showed the highest dehiscence rates in both flap types, whereas ventral body and perineal flaps demonstrated lower dehiscence rates. Passive drains showed higher dehiscence frequencies than active drainage systems. Necrosis was strongly associated with wound dehiscence in both APFs and SPFs, while edema and discharge were additionally associated with dehiscence in APFs and SPFs, respectively. Preoperative hematologic and biochemical abnormalities, body weight, sex, age, and granulation tissue were not significantly associated with surgical outcomes.

**Conclusion:**

APFs and SPFs demonstrated comparable clinical outcomes and complication rates in canine reconstructive surgery. Postoperative complications, particularly necrosis, were strongly associated with wound dehiscence. Careful flap selection, meticulous surgical technique, appropriate drainage management, and close postoperative monitoring are essential to optimize reconstructive outcomes, especially in anatomically challenging regions such as the distal limbs.

## INTRODUCTION

Axial pattern flaps (APFs) and subdermal plexus flaps (SPFs) are two fundamental reconstructive techniques used to manage extensive skin defects in dogs, particularly when primary closure techniques are not achievable [1]. APFs are generally considered more reliable because they incorporate a direct cutaneous artery and vein, which provide a robust and predictable vascular supply. This vascular advantage allows APFs to support longer and narrower flap designs, making them particularly useful for large or anatomically challenging defects [2, 3]. In contrast, SPFs rely primarily on the subdermal vascular plexus, in which blood flow is supplied through a random network of small cutaneous vessels. Consequently, SPFs are considered to have a less predictable blood supply and may carry a higher risk of distal ischemia and necrosis [4]. Nevertheless, SPFs remain widely used because they are technically simpler, require less extensive dissection, and are suitable for many reconstructive situations [1, 4]. Furthermore, widening the base of an SPF may inadvertently include adjacent direct cutaneous vessels in certain anatomical regions [4], thereby reducing the practical distinction between APFs and SPFs in some clinical settings.

Successful skin flap reconstruction depends on multiple interacting clinical and surgical factors [5–7]. Previous studies have suggested that wound etiology, anatomical location, tissue condition, vascular integrity, and postoperative management may substantially influence flap survival and complication rates [5–8]. Chronic wounds are among the most common indications for APFs because these flaps provide enhanced vascular support to compromised tissues [1, 5]. Conversely, SPFs have been associated with higher complication rates when used in wounds with poor vascularity or extensive tissue damage [6]. Anatomical location is also considered an important determinant of surgical outcome. Distal limb wounds are particularly prone to complications because of limited skin mobility, reduced soft tissue coverage, and relatively compromised blood supply [6, 8]. In addition, postoperative management strategies, particularly drainage methods, may influence wound healing and flap viability. Passive drains have been associated with higher complication rates, whereas active drainage systems may reduce dead space and fluid accumulation, thereby improving healing outcomes [9, 10]. Complications that compromise vascular perfusion, including edema, infection, hematoma formation, and necrosis, are also likely to adversely affect flap survival and increase the risk of wound dehiscence.

Despite the widespread clinical use of APFs and SPFs in canine reconstructive surgery, direct comparative evidence between these techniques remains limited. Most previous studies have evaluated APFs or SPFs independently rather than within the same study population [5–7]. In addition, several studies have combined dogs and cats in their analyses, which may introduce species-related variability and limit interpretation of canine-specific outcomes [5, 6]. Consequently, the comparative performance of APFs and SPFs in dogs remains incompletely understood.

Furthermore, previous investigations have primarily focused on overall complication rates or flap survival, with relatively limited evaluation of the broader clinical factors that may influence wound healing outcomes. Variables such as patient demographics, hematologic and biochemical abnormalities, wound location, drainage methods, and postoperative complications have not been comprehensively compared between APFs and SPFs in a single canine population. In particular, the relationship between these factors and wound dehiscence has not been clearly established. Therefore, it remains uncertain whether the theoretical vascular advantage of APFs translates into superior clinical outcomes compared with SPFs under routine clinical conditions.

Thus, the objective of the present study was to retrospectively compare APFs and SPFs in dogs by evaluating flap usage patterns, patient characteristics, preoperative hematologic and biochemical parameters, surgical indications, wound locations, drainage methods, postoperative complications, and factors associated with wound dehiscence. We hypothesized that APFs would demonstrate comparable wound dehiscence and complication rates to SPFs and that clinical and surgical factors, including patient characteristics, preoperative hematologic and biochemical abnormalities, wound location, drainage method, and postoperative complications, would significantly influence surgical outcomes.

## MATERIALS AND METHODS

### Ethical approval

This retrospective study was reviewed and approved by the Institutional Animal Care and Use Committee of Kasetsart University (approval no. ACKU68-VET-094). All procedures adhered to the ethical standards for animal use in research established by the National Research Council of Thailand (license no. U1-09430-2564). The study was based solely on retrospective evaluation of existing medical records. No experimental procedures, invasive interventions, or direct animal handling were performed specifically for research purposes. Therefore, no additional risks were imposed on the animals. All dogs received routine clinical care at the Kasetsart University Veterinary Teaching Hospital according to institutional clinical protocols.

### Study period and location

This retrospective study was conducted using medical records from dogs that underwent APF or SPF reconstruction at the Kasetsart University Veterinary Teaching Hospital, Bangkhen Campus, Bangkok, Thailand, between July 15, 2013, and July 14, 2025.

### Study design and case selection

**Figure 1:**
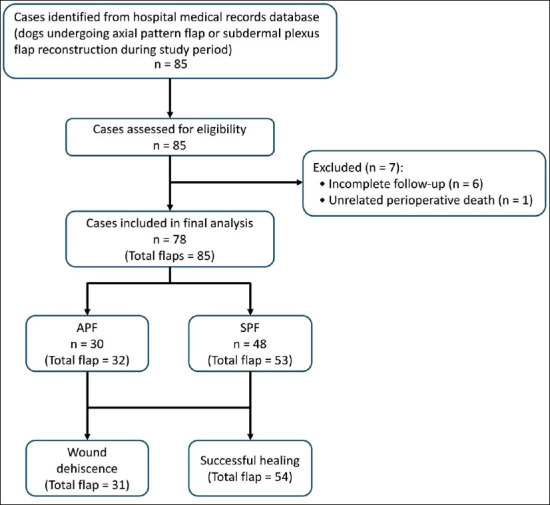
Flow diagram of case selection for dogs undergoing axial pattern flap (APF) or subdermal plexus flap (SPF) reconstruction. Cases were retrospectively identified from the hospital medical record database and included as a consecutive case series. Cases were excluded because of incomplete follow-up or perioperative death before outcome assessment.

Cases were identified from the hospital medical record database, and all eligible cases during the study period were included, representing a consecutive case series. Cases were included if complete medical records and sufficient postoperative follow-up information were available to assess wound healing outcomes. Cases were excluded if follow-up information was insufficient to determine wound outcome, including loss to follow-up before suture removal or absence of documented wound assessment, or if perioperative death occurred before postoperative outcome evaluation. Incomplete follow-up was defined as inadequate postoperative documentation to classify the outcome as either successful healing or wound dehiscence. A flow diagram summarizing case identification, exclusion, and inclusion is presented in Figure 1.

### Data collection

Data were retrospectively extracted from medical records by the investigators. The collected variables included patient demographics, preoperative hematologic and biochemical parameters, preoperative presence of granulation tissue, flap type and subtype, wound location, clinical indication for flap use, drainage method, postoperative complications, and wound healing outcomes. Preoperative laboratory values were obtained within 10 days before surgery for each flap procedure.

Clinical variables, including the presence of granulation tissue and postoperative complications such as discharge, edema, necrosis, and bruising, were recorded according to the attending clinician’s documentation and were not independently reassessed. Granulation tissue was recorded as a binary variable (present or absent) based on preoperative clinical records. Preoperative laboratory values were categorized as normal or abnormal according to institutional reference intervals.

Acute wounds were defined as traumatic injuries evaluated in the hospital emergency unit and surgically managed without prior wound treatment. Chronic wounds were defined as lesions in which previous wound treatments or surgeries had failed before referral or where wound healing had not progressed within the expected timeframe. Chronic dermatitis excisions were categorized according to histopathological confirmation of the excised lesion.

A total of 85 dogs were initially identified. Seven dogs were excluded because of incomplete follow-up (n = 6) or unrelated perioperative death before outcome assessment (n = 1), resulting in 78 dogs included in the study population. The study population comprised 40 males and 38 females with a mean age of 115 ± 44 months. The median body weight was 15.9 kg (range: 2.6–51.5 kg). Five dogs underwent two flap procedures and one dog underwent three flap procedures. Consequently, 85 flap procedures were included in the final analysis.

**Figure 2:**
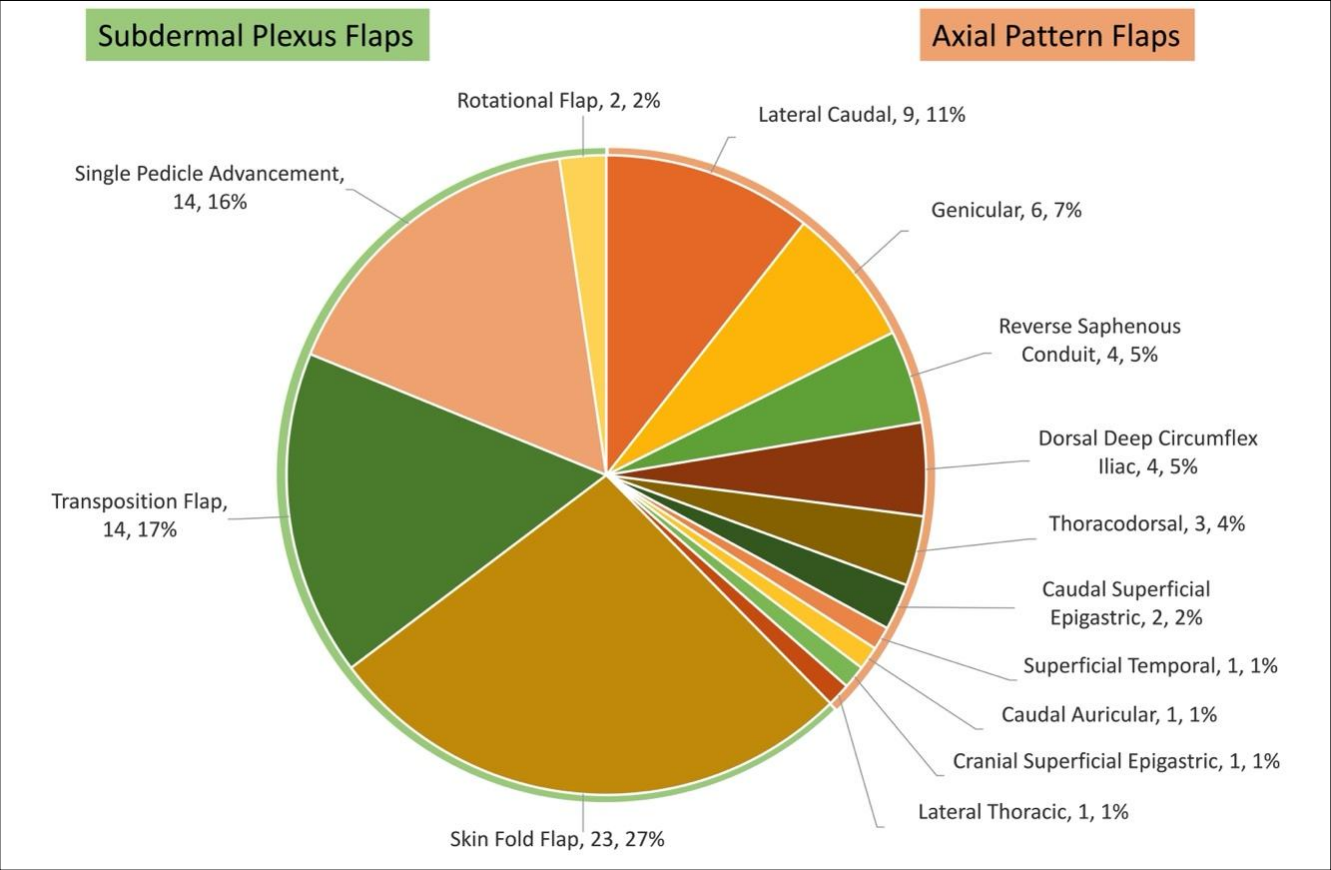
Distribution of skin flap subtypes used for reconstruction in dogs. Subdermal plexus flaps (SPFs) and axial pattern flaps (APFs) are presented as proportions of the total number of flaps (n = 85). Each segment represents a specific flap subtype, with the corresponding number of cases and percentage indicated.

Of the 85 flaps analyzed, 53 were SPFs (62.4%) and 32 were APFs (37.6%) (Figure 2). Among SPFs, skin fold flaps predominated (n = 23), particularly flank fold flaps (n = 20). Among APFs, the lateral caudal APF was the most frequently used subtype (n = 9), followed by genicular flaps (n = 6) and deep circumflex iliac dorsal branch flaps (n = 5).

The APF group comprised 30 dogs. Mixed-breed dogs were most common (n = 12; 40.0%), followed by Poodles (n = 5; 16.7%). Other breeds included Chihuahua, Cocker Spaniel, Dalmatian, Golden Retriever, Labrador Retriever, Pug, Schnauzer, Shih Tzu, Siberian Husky, and Thai Bangkaew, each represented by one or two dogs. The median body weight in the APF group was 14.0 kg (range: 3.18–45.9 kg).

The SPF group comprised 48 dogs. Mixed-breed dogs were also the most common breed (n = 16; 33.3%), followed by Shih Tzus (n = 6; 12.5%), Poodles (n = 4; 8.3%), Labrador Retrievers (n = 4; 8.3%), and Golden Retrievers (n = 3; 6.3%). Additional breeds included Akita, Alaskan Malamute, Beagle, Chihuahua, Dalmatian, French Bulldog, German Shepherd, Jack Russell Terrier, Miniature Pinscher, Pomeranian, Siberian Husky, and Thai Bangkaew, each represented by one or two dogs. The median body weight in the SPF group was 17.5 kg (range: 2.6–51.5 kg).

### Skin flap procedures

Flaps were classified as SPFs or APFs according to vascular supply. APFs were defined as flaps incorporating a direct cutaneous artery and vein, whereas SPFs relied on the subdermal vascular plexus without a named direct cutaneous vessel. Flap classification was determined from anatomical location and flap design documented in the surgical reports.

Both SPF and APF procedures were performed according to standard reconstructive techniques described by Kirpensteijn and ter Haar [10]. All flaps were maintained on a pedicle at the donor site. In dogs with multiple wound sites, each flap was evaluated separately, resulting in a total of 85 flap procedures included in the analysis.

All procedures were performed under standard aseptic surgical conditions according to institutional protocols. Anesthetic, analgesic, and antimicrobial protocols were determined by the attending anesthesiologist and surgeon based on individual patient requirements. Standard reconstructive surgical principles, including preservation of vascular supply, atraumatic tissue handling, and tension-minimized closure, were applied in all procedures.

Drains were categorized as passive drains (e.g., Penrose drain), commercial active drains (e.g., Jackson-Pratt drain), or homemade active drains, as previously described by Campbell [11]. The selection of flap type, drainage method, and duration of drain placement were determined according to surgeon preference, intraoperative findings, anticipated dead space, and clinical judgment.

### Complications and outcome assessment

Postoperative complications were defined as adverse events documented during hospitalization or follow-up visits. Follow-up evaluations were conducted from the day of surgery until suture removal or until postoperative complications developed and resolved, up to 6 months postoperatively. A minimum follow-up period sufficient to determine wound healing outcome was required for case inclusion.

Outcomes were classified as either wound dehiscence or successful healing. Wound dehiscence was defined as partial or complete separation of flap edges or the presence of flap necrosis requiring debridement. When possible, dehiscence was further characterized as partial dehiscence or more severe dehiscence involving flap edge necrosis requiring surgical intervention. The requirement for additional surgical management was also recorded. Successful healing was defined as complete flap healing without dehiscence or necrosis.

Additional postoperative complications included edema, wound discharge, necrosis, and bruising. Surgical site infections were identified according to clinical documentation in the medical records, including purulent discharge, local inflammation, and/or positive bacterial culture results when available. Because of the limited number of cases, surgical site infections were reported descriptively and excluded from statistical analyses.

The unit of analysis was defined at the flap level, with each flap treated as an independent observational unit representing a separate surgical procedure.

### Statistical analysis

Statistical analyses were performed using Stata version 12.1 (StataCorp, College Station, TX, USA). Continuous variables were assessed for normality using the Shapiro–Wilk test. Normally distributed variables were compared using the independent t-test, whereas non-normally distributed variables were analyzed using the Mann–Whitney U test. Categorical variables were summarized as frequencies and percentages.

Associations between clinical variables and wound dehiscence were evaluated using Fisher’s exact test. Because of the exploratory nature of this retrospective study and the relatively limited sample size, multivariate logistic regression analysis was not performed, and the analyses were limited to univariate comparisons. A p < 0.05 was considered statistically significant.

## RESULTS

A total of 85 flap procedures were performed in 78 dogs, including a small number of dogs that underwent multiple flap procedures. The general patient characteristics, preoperative presence of granulation tissue, and postoperative complications are summarized in Table 1 and stratified according to wound outcome (wound dehiscence vs. successful healing). Thirty-one flaps (36.5%) were classified in the wound dehiscence group, whereas 54 flaps (63.5%) achieved successful healing.

**Table 1:** General characteristics of dogs undergoing skin flap surgery and comparison between wound dehiscence and successful healing groups.

Variable	Wound dehiscence (n = 31)	Successful healing (n = 54)	p-value
Mean age (months)	117 ± 43	115 ± 46	0.887
Body weight (kg), median (range)	14.0 (3.18–45.9)	17.5 (2.6–51.5)	0.454
Sex			0.260
Male	19 (61.29%)	25 (46.30%)	
Female	12 (38.71%)	29 (53.70%)	
Granulation tissue			0.739
Yes	3 (9.68%)	8 (14.81%)	
No	28 (90.32%)	46 (85.19%)	
Discharge			0.002
Yes	6 (19.35%)	0 (0.00%)	
No	25 (80.65%)	54 (100.00%)	
Edema			0.049
Yes	16 (51.61%)	15 (27.78%)	
No	15 (48.39%)	39 (72.22%)	
Necrosis			<0.001
Yes	24 (77.42%)	4 (7.41%)	
No	7 (22.58%)	50 (92.59%)	
Bruising			0.248
Yes	15 (48.39%)	18 (33.33%)	
No	16 (51.61%)	36 (66.67%)	

There was no significant difference in mean age between the wound dehiscence and successful healing groups. Similarly, body weight did not differ significantly between dogs with wound dehiscence and those with successful healing. No significant associations were identified between wound outcome and sex distribution or the preoperative presence of granulation tissue.

Postoperative complications included wound discharge (n = 6), edema (n = 31), necrosis (n = 28), and bruising (n = 33). Several complications were more frequently observed in the wound dehiscence group. Necrosis showed a strong association with wound dehiscence (OR: 42.86; 95% CI: 11.8–155.6). Edema was also associated with increased odds of dehiscence (OR: 2.77; 95% CI: 1.07–7.17). Wound discharge demonstrated a similar trend; however, the estimate was imprecise because of the limited number of affected cases (OR: 28.1; 95% CI: 1.5–521).

Postoperative infections occurred in two flaps (2.4%) from two dogs in which *Escherichia coli* was isolated. Both cases involved lateral caudal APFs located in the perineal region following mass excision. These cases were managed with antimicrobial therapy based on antimicrobial susceptibility testing, debridement, local wound care, and medical management until complete healing was achieved without additional surgical closure.

### Comparison of wound dehiscence between APF_S_ and SPF_S_

Wound dehiscence was observed in 12 of 32 APFs (37.5%) and 19 of 53 SPFs (35.8%). Overall, the incidence of wound dehiscence did not differ significantly between the two flap types (p = 1.000). The odds ratio for wound dehiscence in APFs compared with SPFs was 1.07 (95% CI: 0.43–2.67), indicating no significant difference between groups. Among the 31 flaps with wound dehiscence, 28 (90.3%) were classified as partial dehiscence, whereas 3 (9.7%) involved flap edge necrosis requiring debridement. Most cases (28/31; 90.3%) were successfully managed with medical treatment and local wound care. Revision surgery was required in three cases (9.7%), all of which were managed using simple appositional closure without the need for additional flap reconstruction. There was no significant difference in body weight between dogs treated with APFs and SPFs (p = 0.454). Within each flap type, body weight was not significantly associated with wound outcome (APFs: p = 0.186; SPFs: p = 0.339).

### Preoperative hematologic and biochemical parameters

Preoperative hematologic and biochemical parameters are summarized in Table 2. When analyzed as continuous variables, no significant differences were observed between flap types or between wound outcome groups (all p > 0.05).

**Table 2:** Preoperative hematologic and biochemical profiles in dogs undergoing skin flap reconstruction.

Parameter	Reference interval	Axial pattern flaps	Subdermal plexus flaps	p-value	Successful healing	Dehiscence	p-value
Hematocrit	30–57	40.7 ± 5.9 (28.7–56.3)	38.8 ± 6.8 (22.7–51.5)	0.317	40.2 ± 6.4 (22.7–56.3)	38.7 ± 5.8 (28.7–49.2)	0.323
White blood cell count (×10³/µL)	5–15	12.9 (6.05–33.55)	14.2 (5.59–25.78)	0.546	13.1 (5.59–33.55)	14.8 (6.35–25.78)	0.399
Platelet count (×10³/µL)	200–620	318 (130–620)	295 (150–598)	0.488	310 (130–620)	290 (180–520)	0.648
Albumin (g/dL)	2.3–3.2	2.9 (2.0–3.2)	2.8 (1.9–3.2)	0.734	2.9 (1.9–3.2)	2.7 (2.0–3.2)	0.434
Creatinine (mg/dL)	0.5–1.7	0.88 (0.5–2.45)	0.99 (0.52–1.80)	0.148	0.92 (0.51–2.45)	0.98 (0.60–1.80)	0.306
Alanine transaminase activity (U/L)	10–109	36 (12–439)	42 (15–210)	0.259	37 (12–439)	41 (18–210)	0.723

Data are presented as mean ± standard deviation or median (range). Comparisons between groups were performed using the Mann–Whitney U test.

Similarly, when laboratory parameters were categorized as abnormalities according to reference intervals (Table 3), anemia, thrombocytopenia, leukocytosis, hypoalbuminemia, elevated creatinine concentration, and elevated alanine transaminase activity were not significantly associated with wound healing outcome or wound dehiscence in either APFs or SPFs.

**Table 3:** Preoperative hematologic and biochemical abnormalities in dogs undergoing skin flap reconstruction.

Variable	Axial pattern flaps		p-value	Subdermal plexus flaps		p-value
	
	Successful healing (n = 20)	Dehiscence (n = 12)		Successful healing (n = 34)	Dehiscence (n = 19)	
Anemia (PCV <30%; n = 85)			1.000			1.000
Yes	0	0		2	1	
No	20	12		32	18	
Thrombocytopenia (<2 × 10⁵/µL; n = 85)			0.375			1.000
Yes	0	1		3	2	
No	20	11		31	17	
Leukocytosis (>15 × 10³/µL; n = 85)			0.696			0.380
Yes	5	4		10	8	
No	15	8		24	11	
Hypoalbuminemia (<2.3 g/dL; n = 70)			1.000			1.000
Yes	3	2		3	1	
No	15	7		25	14	
Elevated creatinine (>1.7 mg/dL; n = 85)			0.375			1.000
Yes	0	1		1	0	
No	20	11		33	19	
Elevated ALT level (>109 U/L; n = 84)			1.000			1.000
Yes	1	0		3	2	
No	19	12		31	16	

ALT = Alanine transaminase, PCV = Packed cell volume.

### Surgical indications

A comparison of surgical indications between the APF and SPF groups is summarized in Table 4. Mass removal was the most common indication overall (69/85; 81.2%), with comparable wound dehiscence rates between APFs and SPFs (approximately 36%). In all cases involving mass removal, flap reconstruction was performed during the same surgical procedure as tumor excision.

**Table 4:** Incidence of wound dehiscence in dogs undergoing axial pattern flaps and subdermal plexus flaps according to surgical indication.

Indication for flap use	Axial pattern flaps		Subdermal plexus flaps		p-value

	Total (n = 32)	Dehiscence (n = 12)	Total (n = 53)	Dehiscence (n = 19)	
Mass removal	30 (93.75%)	11 (36.67%)	39 (73.58%)	14 (35.89%)	0.948
Acute wound closure	0 (0.00%)	0 (0.00%)	4 (7.55%)	2 (50.00%)	–
Chronic wound closure	2 (6.25%)	1 (50.00%)	9 (16.98%)	2 (22.22%)	0.425
Chronic dermatitis	0 (0.00%)	0 (0.00%)	1 (1.89%)	1 (100.00%)	–

Other indications included chronic wound closure (11/85; 12.9%), acute wound closure (4/85; 4.7%), and chronic dermatitis excision (1/85; 1.2%). Based on available medical records, acute wounds (n = 4) were surgically managed approximately 6–48 h after injury. In contrast, chronic wounds (n = 11) had a median duration of 44 days (range: 26–300 days) before flap reconstruction and had generally undergone previous wound management procedures.

### Wound locations

The 85 skin flaps were distributed across multiple anatomical regions, including the head and neck (16/85; 18.8%), dorsal body (12/85; 14.1%), ventral body (18/85; 21.2%), proximal limb (18/85; 21.2%), distal limb (15/85; 17.6%), and perineum (6/85; 7.1%).

When stratified according to anatomical location (Table 5), distal limb flaps exhibited the highest wound dehiscence rates in both APFs and SPFs. Wound dehiscence was also observed in the head and neck, dorsal body, and proximal limb regions in both flap groups. In contrast, ventral body and perineal flaps demonstrated lower dehiscence rates. No wound dehiscence occurred in APFs located on the ventral body.

**Table 5:** Incidence of wound dehiscence in dogs undergoing axial pattern flaps and subdermal plexus flaps according to anatomical location.

Skin flap location	Axial pattern flaps		Subdermal plexus flaps		p-value

	Total (n = 32)	Dehiscence (n = 12)	Total (n = 53)	Dehiscence (n = 19)	
Head and neck	2 (6.25%)	1 (50.00%)	14 (26.42%)	5 (35.71%)	0.696
Dorsal body	5 (15.62%)	1 (20.00%)	7 (13.21%)	3 (42.85%)	0.408
Ventral body	2 (6.25%)	0 (0.00%)	16 (30.19%)	4 (25.00%)	0.423
Proximal limb	8 (25.00%)	3 (37.50%)	10 (18.87%)	4 (40.00%)	0.914
Distal limb	9 (28.12%)	6 (66.67%)	6 (11.32%)	3 (50.00%)	0.519
Perineum	6 (18.75%)	1 (16.67%)	0 (0.00%)	0 (0.00%)	–

### Drainage methods

Surgical drains were used in 54 of 85 flaps (63.5%). Passive drains were placed in 19 of 54 flaps (35.2%) and remained in place for a median duration of 8 days (range: 4–15 days), including 7 days (range: 5–11 days) for APFs and 9 days (range: 4–15 days) for SPFs.

Homemade active drains were used in 22 of 54 flaps (40.7%) and remained in place for a median duration of 9 days (range: 2–19 days), including 9 days (range: 4–10 days) for APFs and 8 days (range: 2–19 days) for SPFs.

Commercial active drains were used in 13 of 54 flaps (24.1%) and remained in place for a median duration of 6 days (range: 4–12 days), including 6 days (range: 5–6 days) for APFs and 7.5 days (range: 4–12 days) for SPFs.

No significant differences were observed between APFs and SPFs regarding the duration of passive drain placement (p = 0.243), homemade active drain placement (p = 0.613), or commercial active drain placement (p = 0.346). Furthermore, the incidence of wound dehiscence did not differ significantly between APFs and SPFs when stratified according to drainage type (Table 6; all p > 0.05).

**Table 6:** Incidence of wound dehiscence in dogs undergoing axial pattern flaps and subdermal plexus flaps according to drainage method.

Drainage method	Axial pattern flaps		Subdermal plexus flaps		p-value

	Total (n = 32)	Dehiscence (n = 12)	Total (n = 53)	Dehiscence (n = 19)	
No drain	12 (37.50%)	5 (41.67%)	19 (35.85%)	6 (31.58%)	0.568
Passive drain	7 (21.88%)	4 (57.14%)	12 (22.64%)	7 (58.33%)	0.960
Active drain (homemade)	10 (31.25%)	3 (30.00%)	12 (22.64%)	2 (16.67%)	0.457
Active drain (commercial)	3 (9.38%)	0 (0.00%)	10 (18.87%)	4 (40.00%)	0.188

**Table 7:** Association between postoperative complications and wound healing outcomes in dogs undergoing axial pattern flaps and subdermal plexus flaps.

Complication	Axial pattern flaps		p-value	Subdermal plexus flaps		p-value
	
	Successful healing (n = 20)	Dehiscence (n = 12)		Successful healing (n = 34)	Dehiscence (n = 19)	
Discharge			0.133			0.005
Yes	0 (0.00%)	2 (16.67%)		0 (0.00%)	4 (21.05%)	
No	20 (100.00%)	10 (83.33%)		34 (100.00%)	15 (78.95%)	
Edema			0.002			0.770
Yes	4 (20.00%)	9 (75.00%)		11 (32.35%)	7 (36.84%)	
No	16 (80.00%)	3 (25.00%)		23 (67.65%)	12 (63.16%)	
Necrosis			0.002			<0.001
Yes	3 (15.00%)	9 (75.00%)		1 (2.94%)	15 (78.95%)	
No	17 (85.00%)	3 (25.00%)		33 (97.06%)	4 (21.05%)	
Bruising			0.144			0.770
Yes	7 (35.00%)	8 (66.67%)		11 (32.35%)	7 (36.84%)	
No	13 (65.00%)	4 (33.33%)		23 (67.65%)	12 (63.16%)	

## DISCUSSION

### Principal findings

This study evaluated patient characteristics, preoperative hematologic and biochemical parameters, surgical indications, wound locations, drainage methods, and postoperative complications in dogs undergoing skin flap reconstruction. To the authors’ knowledge, this is the first retrospective study to compare these factors between APFs and SPFs, as well as between wound dehiscence and successful healing outcomes, in a canine population.

The findings should be interpreted with caution because the analysis was limited to univariate comparisons. Therefore, the observed associations may have been influenced by confounding factors and should not be interpreted as independent predictors. Surgical outcomes are multifactorial, and factors such as wound location, flap type, drainage method, wound condition, and patient characteristics are likely interrelated.

### Patient characteristics and preoperative laboratory parameters

Age and sex did not differ significantly between dogs that developed wound dehiscence and those that healed successfully in either the APF or SPF groups. These demographic variables were not clearly associated with flap outcome in this study. Although increased body weight has been reported as a potential risk factor for postoperative complications in dogs [7], body weight was not significantly associated with wound outcome in the present study.

Preoperative hematologic and biochemical parameters were evaluated both as continuous variables and as categorical abnormalities based on institutional reference intervals. Neither approach demonstrated a significant association with wound outcome. Although categorization may better reflect clinically relevant thresholds, analysis of continuous values provides additional information on the magnitude and variability of these parameters. The consistency of findings across both approaches suggests that the evaluated preoperative hematologic and biochemical parameters had limited predictive value for flap healing outcomes in this study population.

### Comparison between APFS and SPFS

APFs are traditionally considered more reliable in small animal reconstructive surgery because of their direct cutaneous arterial supply [1]. Therefore, APFs are often expected to have higher survival rates than SPFs. However, in the present study, wound dehiscence did not differ significantly between APFs and SPFs (37.5% vs. 35.8%). Overall complication rates were also comparable between APFs and SPFs (65.6% vs. 58.5%), with an overall complication rate of 61.0%. Similarly, a recent study of 52 cats undergoing 62 skin flaps (30 APFs and 32 SPFs) found no significant difference in complication rates between APFs and SPFs, supporting the notion that flap type alone may not be the primary determinant of surgical outcome [12].

These findings are broadly consistent with previously reported complication ranges, although considerable variation exists among studies. Reported dehiscence rates for APFs in dogs have reached up to 58%, with overall complication rates approaching 80% [5]. In contrast, SPFs have been associated with dehiscence and overall complication rates of approximately 30% and 51%, respectively [6]. However, some previous studies included both dogs and cats, which may have introduced variation due to mixed-species populations [5, 6]. More recent canine-specific data reported complication rates of 53.6% in SPFs [7].

Despite the theoretical vascular advantage of APFs, the comparable outcomes observed in this study suggest that factors other than flap type may play a greater role in determining clinical outcome. In clinical practice, APFs are often selected for larger or more complex defects, whereas SPFs are typically used for smaller or less complex wounds. Because wound size and defect complexity were not consistently documented in this retrospective study, APF cases may have been inherently more challenging, which may have influenced the observed outcomes.

### Possible biological and surgical explanations

Biological mechanisms such as hypoxia-induced angiogenesis have been proposed as contributors to flap survival, although these mechanisms were not evaluated in the present study. Transient local hypoxia after flap transposition may stimulate angiogenesis through pathways involving hypoxia-inducible factor-1α and vascular endothelial growth factor [13]. Several studies have investigated methods to enhance angiogenesis and improve skin flap survival [14]. However, the relevance of these mechanisms to the present findings remains uncertain and should be considered speculative.

Other factors, including careful flap design, appropriate case selection, meticulous surgical technique, preservation of vascular supply, atraumatic tissue handling, and appropriate perioperative care, may have contributed to favorable outcomes, particularly in SPFs.

### Postoperative complications and wound dehiscence

Postoperative complications were more frequent in the wound dehiscence group, particularly discharge, edema, and necrosis. Bruising was also more common in the dehiscence group, although this association was not statistically significant. Adverse events that compromise skin blood supply, such as excessive tension, infection, hematoma, edema, or vascular injury, may impair perfusion and increase the risk of poor postoperative outcomes.

Necrosis was more frequently observed in cases with wound dehiscence across both APFs and SPFs. This finding is consistent with previous studies that described necrosis as a common and clinically important complication of reconstructive flap surgery [1, 15]. Necrosis may result from compromised vascularity, excessive tension, infection, or direct trauma to the vascular pedicle [1]. Technical factors should also be considered because inaccurate assessment of vascular anatomy or failure to preserve cutaneous vessels may contribute to flap necrosis.

Near-infrared angiography has recently been introduced for intraoperative vascular assessment in canine reconstructive surgery [16, 17]. This technique uses indocyanine green to provide real-time visualization of arterial supply and flap perfusion [16]. Therefore, it may help surgeons identify viable flap margins and evaluate vascular supply during surgery. Although necrosis appeared to be associated with wound dehiscence in this study, edema and discharge were also more frequently observed in affected cases. These findings may indicate compromised healing; however, this relationship was not directly evaluated and should be interpreted with caution.

### Surgical indications and wound condition

The primary indication for flap reconstruction in both APFs and SPFs was wound closure following mass removal, which is consistent with previous studies [6, 18–20]. Other indications included acute wound closure, chronic wound closure, and chronic dermatitis excision. APFs were not used for acute wound closure or chronic dermatitis in this study. This may reflect case distribution and surgeon preference within the study population.

A greater proportion of APFs was used after tumor excision, possibly because larger masses required wide excision to reduce recurrence risk. APFs are particularly suitable for such wounds because their robust vascular supply can support larger skin flaps [1]. Acute wounds reconstructed with SPFs showed a high dehiscence rate, likely because traumatic wounds are commonly associated with devitalized tissue, vascular injury, and contamination, all of which may impair wound healing [8]. Therefore, severely contaminated or compromised wounds should be adequately managed before flap reconstruction whenever possible.

Chronic wound closure with APFs also showed a high dehiscence rate. This may be related to the chronic nature of these wounds. Chronic wounds often involve persistent inflammation, bacterial infection, and biofilm formation, which can impair normal tissue repair [21, 22].

### Effect of wound location

Wound location appeared to influence surgical outcomes. Distal limb flaps had the highest dehiscence rates in both APFs and SPFs. This may be explained by relatively poor perfusion, high mobility, limited soft tissue coverage, and increased tension in the distal extremities [23, 24]. Joint movement and pressure during ambulation may further compromise flap survival. These findings are consistent with previous studies reporting high complication and dehiscence rates in distal limb reconstruction [6, 25].

Conversely, lower complication rates were observed in flaps located on the ventral body and perineal region. These areas generally have better vascular supply and lower mechanical strain [2, 26]. However, the perineal region is prone to fecal and urinary contamination, which may increase the risk of surgical site infection, as observed in this study. Overall, these findings suggest that recipient site characteristics, especially regional blood supply and tissue tension, play an important role in flap success.

### Drainage method and postoperative care

Passive drains had the highest dehiscence rates in both APFs and SPFs. Passive drains are prone to ascending infection and local skin irritation [10], which may contribute to impaired healing. In contrast, homemade and commercial active drainage systems showed lower dehiscence rates, likely because they more effectively manage dead space and fluid accumulation.

Homemade active drains may represent a low-cost closed-suction option and showed favorable outcomes in this study. Commercial active drains were associated with 0% dehiscence in APFs, whereas SPFs treated with commercial active drains had higher dehiscence rates. This difference may have resulted from case selection, wound condition, surgical technique, drain placement, or postoperative care. Although active drainage systems may reduce hospitalization time and cost, recent studies have shown that active drain placement in outpatient cases may increase minor complications, such as tube dislodgement or discharge [9, 27]. Therefore, drain care should be considered an important component of postoperative management. However, these findings should be interpreted as exploratory because drainage selection was not randomized and may have been influenced by case complexity.

### Clinical implications

An important source of potential bias in this study was surgeon-dependent decision-making in flap selection and drainage use. In clinical practice, these decisions are influenced by wound size, wound location, tissue condition, surgeon experience, and anticipated postoperative complications. Therefore, APFs and SPFs may not have been used in directly comparable cases. This selection bias may partly explain the similar complication rates between APFs and SPFs.

From a clinical perspective, these findings may support a practical risk-based approach to flap surgery in dogs. Although no single factor was identified as an independent predictor, several variables were more frequently observed in cases with wound dehiscence. These included distal limb reconstruction, acute or chronic wounds, and postoperative complications such as necrosis, edema, or discharge. Lower-risk cases may include flaps in well-vascularized regions, such as the ventral body or perineum, without significant postoperative complications. Higher risk cases may include anatomically challenging locations, compromised wound conditions, or early postoperative signs of impaired healing. This approach may assist surgeons in flap selection, perioperative planning, and closer postoperative monitoring.

### Study limitations and future directions

This study had several limitations, mainly related to its retrospective design. The lack of standardized preoperative and perioperative protocols, postoperative care, and follow-up schedules limited control over potential confounding factors. Treatment decisions were also based on individual surgeon preference rather than standardized criteria, introducing potential selection bias.

The absence of multivariate analysis limited the ability to identify independent predictors of wound dehiscence. Advanced approaches such as propensity score matching or stratified analysis were not feasible because of the limited sample size. Therefore, residual confounding cannot be excluded.

Important surgical variables, including wound size, defect complexity, flap dimensions, length-to-width ratio, arc of rotation, and objective assessment of tissue tension, were not consistently documented and therefore could not be analyzed. These factors may represent important unmeasured confounders because they often influence flap selection and surgical outcomes. Objective assessment of flap perfusion, such as near-infrared angiography, was also not performed, limiting evaluation of vascular factors associated with flap survival.

Additional adverse events, including seroma, hematoma, pain scores, systemic complications, and concurrent diseases, were not consistently recorded in the medical records and could not be systematically analyzed. Some dogs contributed more than one flap to the dataset, and each flap was treated as an independent observational unit. Therefore, potential non-independence of observations was not accounted for statistically.

The generalizability of the findings should also be considered. This study was conducted at a single university veterinary teaching hospital, and the results may reflect the specific case mix, surgical practices, and expertise within this setting. Regional factors, including climate, wound management practices, and husbandry conditions, may also influence surgical outcomes and limit direct comparison with other populations.

Future prospective multicenter studies with standardized protocols, larger sample sizes, multivariate analyses, and comprehensive assessment of functional and cosmetic outcomes are needed to clarify the comparative performance of APFs and SPFs in canine reconstructive surgery.

## CONCLUSION

Both APFs and SPFs were effective reconstructive techniques in dogs and demonstrated comparable rates of wound dehiscence and postoperative complications. Although APFs are traditionally considered to provide superior vascular support, flap type alone did not appear to be the primary determinant of surgical outcome in this study. Wound dehiscence was more frequently associated with postoperative complications, particularly necrosis, and with anatomical location, occurring most frequently in distal limb flaps and least frequently in ventral body and perineal flaps. Acute and chronic wound conditions also appeared to increase the risk of impaired healing.

These findings suggest that factors beyond flap vascular pattern, including wound location and postoperative complications, play important roles in healing outcomes and underscore the importance of careful case selection, meticulous surgical technique, preservation of vascular supply, appropriate drainage management, and close postoperative monitoring to optimize reconstructive outcomes. Clinically, these observations may support a practical risk-based approach in which wound condition, anatomical location, and early postoperative complications are considered during surgical planning and postoperative management.

The present study provides clinically relevant comparative data regarding APFs and SPFs in a canine population and contributes to the limited literature directly evaluating these reconstructive techniques within the same study cohort. However, the retrospective design, limited sample size, absence of multivariate analysis, and lack of standardized surgical and postoperative protocols should be considered when interpreting the findings.

Future prospective multicenter studies with larger populations, standardized protocols, objective perfusion assessment, and multivariate analyses are warranted to better identify independent predictors of flap success and further clarify the comparative performance of APFs and SPFs in canine reconstructive surgery.

## DATA AVAILABILITY

The datasets used and analyzed during the study can be available from the corresponding author on a reasonable request.

## AUTHORS’ CONTRIBUTIONS

NB: Data collection, data analysis, data interpretation, literature search, and drafting of the manuscript. PN and SP: Data collection and review of the manuscript. NT: Study design, data analysis, data interpretation, and review of the manuscript. All authors have read and approved the final manuscript.
